# Frequency of MOG-IgG in cerebrospinal fluid versus serum

**DOI:** 10.1136/jnnp-2021-326779

**Published:** 2021-07-14

**Authors:** Samuel Pace, Michael Orrell, Mark Woodhall, Jacqueline Palace, Maria Isabel Leite, Sarosh R Irani, Patrick Waters, Adam E Handel

**Affiliations:** 1 Medical School, University of Oxford, Oxford, Oxfordshire, UK; 2 Oxford Autoimmune Neurology Group, University of Oxford, Oxford, Oxfordshire, UK; 3 Nuffield Department of Clinical Neurosciences, University of Oxford, Oxford, Oxfordshire, UK

**Keywords:** neuroimmunology, clinical neurology

## Introduction

Immunoglobulin gamma autoantibodies directed against myelin oligodendrocyte glycoprotein (MOG-IgG) are associated with specific neurological syndromes, most frequently acute disseminated encephalomyelitis, optic neuritis and longitudinally extensive transverse myelitis.^1^ For all neurological surface-directed autoantibodies, including MOG-IgG, serum concentrations are higher than cerebrospinal fluid (CSF), consistent with their peripheral generation. Hence, in the UK, CSF testing is not routine in clinical practice. However, recent studies have reported patients with CSF MOG-IgG but without detectable serum MOG-IgG.^2 3^ The prevalence and clinical relevance of this finding is unclear. We aimed to address this by analysing MOG-IgG results in all paired serum and CSF samples from our national testing database.

## Methods

We audited the database of MOG-IgG cell-based assay (CBA) requests from the Oxford Autoimmune Neurology Diagnostic Laboratory between 2011 and 2019. These data encompassed all samples referred to the centre for MOG-IgG testing by physicians over this time period. Serum samples were available from 272 centres, mostly within the UK, and CSF from 97 centres without clear referral bias. MOG-IgG testing was performed using a cell-based assay as previously described, modified to detect MOG-IgG1 since 2014.^4^ CSF was assayed undiluted. Positive or negative MOG-IgG results were available for 22 554 patients; in 533/22 554 (2.4%) the results were in paired serum and CSF (defined as those taken within 31 days of one another). Data were analysed in R with estimated 95% CIs derived from DescTools (Wilson’s method with continuity correction).

For patients with negative serum and positive CSF MOG-IgG results, the patient’s general practitioner and referring neurologists supplied case notes and test results from which pre-specified clinical and paraclinical parameters were extracted (age, gender, clinical manifestations of relapses, prodromal infections, autoimmune personal or family history, malignancy, seizures, MRI findings, CSF results, past and present treatments and disability outcomes). Data were compared with a published cohort.^1^ Our audit protocol was approved by Oxford University Hospitals NHS Foundation Trust (ID 5600).

## Results

Overall, 118/533 (22.1%) patients with paired samples showed MOG-IgG in either serum or CSF of which 66/118 (55.9%) were only positive in the serum and 48/118 (40.7%) were positive in both compartments. We identified only 4/118 (3.4%; 95% CI 1.1% to 9.0%) patients with MOG-IgG exclusively detected in the CSF with semi-quantitative visually assessed CBA scores (1, 1, 2 and 4 out of 4, samples scoring 1 to 1.5 are considered low positive, above 1.5 are considered positive) which were not significantly different to the CBA scores of CSF samples from patients with both serum and CSF MOG-IgG positivity (p=0.53, Wilcoxon rank-sum test).

Clinical details were located on three of the four patients with exclusively CSF MOG-IgG. One patient was diagnosed with pneumococcal meningitis, and neither treated for nor thought to have MOG-IgG-associated disease (CSF CBA Score 1, no follow-up samples). In the remaining two patients (CSF CBA scores 4 and 1), MRI spine results showed longitudinally extensive transverse myelitis (LETM; C2–C5 and T6–T8 in one patient, and from the medulla to conus in the other). In one patient, CSF showed a lymphocytic pleocytosis with slightly elevated protein. CSF oligoclonal bands were negative in both patients. Both patients were treated with a short course of steroids and subsequently with either intravenous immunoglobulins or plasma exchange. We compared their clinical characteristics and outcome measures with a published Oxford cohort of MOG-IgG serum positive patients and found no clearly distinguishing features (table 1).

**Table 1:** A comparison of MOG-IgG CSF+/serum– patient characteristics between this study and the Oxford cohort from Jurynczyk *et al*

Clinical characteristic	MOG-IgG CSF+/serum– (n=2)	Jurynczyk *et al* (n=75)
Age	24, 69	29±16.5 (Mean±SD)
Female (%)	100	56
CSF MOG-IgG CBA score	1, 4	NA
Serum MOG-IgG CBA score	0.75, 0.75	NA
Time since onset (years)	3, 4	NA
Onset attack (%)		
Unilateral ON	0	25
Bilateral ON	0	27
Transverse myelitis	100	20
ADEM	0	20
Simultaneous ON and TM	0	8
Disease course (%)		
Monophasic	100	41
Relapsing	0	59
Endpoints (%)		
VA ≤6/36	0	16
Limiting walking distance	50	7
Permanent bladder dysfunction	50	28
Catheterisation	50	17
Permanent bowel dysfunction	50	20

ADEM, acute disseminated encephalomyelitis; CBA, cell-based assay; scored on a scale of 0 to 4; CSF, cerebrospinal fluid; MOG, myelin oligodendrocyte glycoprotein; NA, non-applicable; ON, optic neuritis; TM, transverse myelitis; VA, visual acuity.

## Discussion

Our study represents an unbiased assessment of all consecutive samples referred to a national laboratory using a highly specific CBA, over 8 years. It shows that MOG-IgG CSF+/serum– patients are detected at a frequency of 0.8% of all paired CSF-serum samples from the UK’s national centre for MOG-IgG testing. Although limited by small numbers, our comparison identified no distinctive clinical features in patients with LETM, suggesting a disease continuum regardless of compartment positivity.^1^

Our findings suggest that, were MOG-IgG testing on CSF to become routine, the number needed to test (NNT) to identify one extra MOG-IgG patient would be 133, assuming the same rate of positivity as seen in the patients with matched serum and CSF results. In actuality, the proportion of positive tests in the cohort with only serum available for testing (n=21 731) was 8.7%, suggesting that the pre-test probability of unselected patients is likely to be lower than for those who underwent paired serum and CSF sampling. Similarly, for patients with only CSF available, the proportion of CSF positive results (6.7%, n=231) was lower than for patients with paired samples (9.8%). Assuming this lower pre-test probability, the NNT to detect one extra case may be as high as 328 if all patients referred for MOG-IgG testing underwent lumbar puncture. Additionally, we identified one patient with pneumococcal meningitis, for whom CSF MOG-IgG was felt to be clinically unrelated to the presentation. This means that the significance of MOG-IgG CSF+/serum– findings must be interpreted within the context of the clinical presentation.^2^ However, our findings should be interpreted in the context of some important limitations.

Limitations include a change in the detection method of MOG-IgG versus IgG1 during the study period (although the proportion of positive MOG-IgG vs IgG1 results did not change significantly, regardless of the method; p=0.1, Fisher’s exact test) and both clinically-relevant CSF+/serum– MOG-IgG patients having monophasic disease after 3–4 years of follow-up. The majority of paired samples were taken on the same day (74%) but it is possible that CSF MOG-IgG positivity could reduce over time. The rate of CSF MOG-IgG positivity in our cohort was lower than that reported in previous studies (71%, n=17; and 92%, n=38).^5 6^ The heterogeneous rates of CSF+/serum– MOG-IgG may be driven by differences in patient recruitment or clinical presentation,^6^ or through differences in the method of measuring CSF MOG-IgG. Although MOG-IgG1 live CBA testing is known to be highly specific for serum MOG-IgG, this is less clear for CSF MOG-IgG and a multicentre study of CSF MOG-IgG testing is an important focus for future research.^4^

Based on our results, extending CSF testing to all patients is likely to only capture a small number of additional patients with additional difficulties in the interpretation of CSF+/serum– MOG-IgG results. Therefore, we suggest CSF MOG-IgG testing should be guided by each individual’s presentation and undertaken when clinically indicated. The benefits of rarely detecting additional MOG-IgG CSF+/serum– patients, with implications for the duration of immunosuppression, would need to be balanced against the small but non-negligible risks of post-lumbar puncture complications. However, it will be important to understand how the specific method used to measure CSF MOG-IgG determines sensitivity and specificity in different patient cohorts. Further research should focus on studying paired serum and CSF samples from patients and controls including individuals with clinically definite multiple sclerosis.

## References

[1] Jurynczyk M , Messina S , Woodhall MR , et al . Clinical presentation and prognosis in MOG-antibody disease: a UK study. Brain 2017;140:3128–38. 10.1093/brain/awx276 29136091

[2] Mariotto S , Gajofatto A , Batzu L , et al . Relevance of antibodies to myelin oligodendrocyte glycoprotein in CSF of seronegative cases. Neurology 2019;93:10.1212/WNL.0000000000008479–72. 10.1212/WNL.0000000000008479 31645473

[3] Aoe S , Kume K , Takata T , et al . Clinical significance of assaying anti-MOG antibody in cerebrospinal fluid in MOG-antibody-associated diseases: a case report. Mult Scler Relat Disord 2019;28:165–6. 10.1016/j.msard.2018.12.035 30605793

[4] Waters PJ , Komorowski L , Woodhall M , et al . A multicenter comparison of MOG-IgG cell-based assays. Neurology 2019;92:10.1212/WNL.0000000000007096–55. 10.1212/WNL.0000000000007096 PMC6511109 30728305

[5] Jarius S , Ruprecht K , Kleiter I , et al . MOG-IgG in NMO and related disorders: a multicenter study of 50 patients. Part 1: frequency, syndrome specificity, influence of disease activity, long-term course, association with AQP4-IgG, and origin. J Neuroinflammation 2016;13:279. 10.1186/s12974-016-0717-1 27788675 PMC5084340

[6] Akaishi T , Takahashi T , Misu T , et al . Difference in the source of Anti-AQP4-IgG and Anti-MOG-IgG antibodies in CSF in patients with neuromyelitis optica spectrum disorder. Neurology 2021. 10.1212/WNL.0000000000012175. [Epub ahead of print: 12 May 2021]. PMC8312856 33980704

